# Asthma and Wheezing Are Associated with Depression and Anxiety in Adults: An Analysis from 54 Countries

**DOI:** 10.1155/2013/929028

**Published:** 2013-03-17

**Authors:** Kai On Wong, Brian Hunter Rowe, Jeroen Douwes, Ambikaipakan Senthilselvan

**Affiliations:** ^1^Department of Public Health Sciences, School of Public Health, University of Alberta, Edmonton, AB, Canada T6G 1C9; ^2^Department of Emergency Medicine, University of Alberta, Edmonton, AB, Canada T6G 2B7; ^3^Centre for Public Health Research, Massey University, Wellington Campus, P.O. Box 756, Wellington, New Zealand

## Abstract

*Background.* Asthma and depression are important public-health concerns worldwide. While some epidemiologic studies have shown asthma and wheezing to be associated with depression and anxiety, the patterns are unclear at the multinational level due to the lack of cross-study comparability. Our study examined the associations of self-reported asthma diagnosis and current wheezing with self-reported depression diagnosis and 30-day anxiety using an international survey. *Methods.* Using the 2002 World Health Survey, a standardized international survey conducted by the WHO, we estimated the associations between diagnosed asthma and current wheezing with diagnosed depression and 30-day anxiety via multiple logistic regressions for 54 countries worldwide. *Results.* Diagnosed depression and 30-day anxiety were associated with diagnosed asthma in 65% and 40% of the countries, respectively. Diagnosed depression and 30-day anxiety were associated with current wheezing in 83% and 82% of the countries, respectively. *Conclusions.* The association between asthma and depression was generally seen at the global level. These results indicated the importance of addressing the asthma-depression comorbidity as public-health and clinical management priorities, in order to improve the overall health of the countries.

## 1. Introduction

Asthma was found to be associated with psychological conditions such as stress, anxiety, and depression in observational studies of various sample populations [1–8]. A meta-analysis was conducted by examining 15 publications of primarily cross-sectional, uncontrolled study designs over a total of 1,494 adults [9]. It found that the overall prevalence of anxiety disorders was generally higher among adults with asthma compared to the general population [9]. The age- and sex-adjusted estimates from a study using the World Mental Health Surveys (WMHS) done by 85,052 adults living in 17 countries across the world have demonstrated the links between asthma diagnosis with anxiety disorders, depressive disorders, and alcohol use disorders [10]. 

Overall, the majority of the epidemiologic studies were limited in geographic coverage, participants' characteristics (such as age), and information available on factors both related to and unrelated to asthma and depression. In addition, most of these studies used different measurement tools to estimate the occurrences and relationships between asthma and depression. As a result, the comparability of these studies was weakened and the ability to make an overall inference regarding the association between asthma and depression at the global level was largely hindered.

The aim of this study was to access the association between asthma and depression on a global scale using a standardized and validated international survey. To accomplish this, we used the World Health Survey (WHS) collected by the World Health Organization (WHO) to assess the existence and nature of the relationships between asthma and psychological conditions.

## 2. Methods

### 2.1. Study Subjects

For this study, we have obtained authorization of usage of the WHS raw data. Ethics approval was obtained from the University of Alberta Health Research Ethics Board (Reference Number: MS1_Pro00003716). The WHO conducted the WHS survey between 2002 and 2004 in 70 countries [11–13]. The WHS was a cross-sectional study with a multistage-stratified, random cluster sampling design which aimed at achieving nationally representative samples while keeping the survey costs efficient. Every eligible individual including all adults aged 18 years and older with a valid home address had a nonzero probability of being selected for participation. Households were randomly chosen first, and then one adult from each household was selected randomly using the Kish Table [11, 13]. Each surveyed participant was assigned a corresponding design weight to account for the level of stratification and clustering applied within the sampling procedures. Fifteen of the 70 countries were excluded from the analysis due to the following reasons: (i) inadequate information on individual design weight; (ii) inadequate information on primary sampling unit (PSU); or (iii) ≥25% of missing data on both primary outcomes: self-reported asthma diagnosis and current wheezing. A total of 258,550 participants from 55 countries were available. The overall household response percentage, calculated as the number of participated household divided by the total number of household invited for study participation in each country, was 87%. Nonresponse was defined as refusal to participate or empty houses. A desired sample size of 5,000 persons per country was recommended by the WHO with exceptions on a by-country basis [11]. 

### 2.2. WHS Questionnaire

The WHS questionnaire was developed in multiple languages using forward-backward translations and cognitive interviews. The survey questionnaire was translated using a translation and linguistic analysis protocol developed by WHO. Forward translation was conducted by a health expert and bilingual group assessing the accuracy and appropriateness of the translations. Next, back-translations were carried out by an independent group of linguistic experts. Each of these expert groups produced a corresponding report on linguistic analysis. The reports were then reviewed and used by WHO to finalize the questionnaire instruments. Cognitive interviews were conducted by pilot-testing the finalized instruments in 100 respondents at each household survey site (ten countries) and 50 respondents were also retested to determine reliability [13]. Analyses were done to determine if the questionnaire was being used and interpreted in a consistent manner across sites which served as an indicator of cross-cultural comparability.

During the phase of actual data collection, participants were interviewed face-to-face or via telephone by trained interviewers who followed standard protocols. The WHS questionnaire had a modular structure for assessing health of individuals in various domains: health system responsiveness, household health care expenditures, and additional modules in areas such as mortality and health state valuations [13, 14]. The health module contained questions on risk factors, diseases, and symptoms and was based on selected domains of the International Classification of Functioning, Disability and Health (ICF) and constructed after reviewing various available assessment tools [13]. Two versions (long and short) of the WHS questionnaire were available [14]. The long version contained additional questions in various modules. Forty-six countries used the long questionnaire and 9 (Australia, Finland, France, Ireland, Israel, Luxembourg, Norway, Portugal, and Sweden) used the short form. 

### 2.3. Dependent Variables: Diagnosed Asthma and Respiratory Symptoms

All of the information on the dependent variables was collected via self-report directly from the participants. The primary dependent variables of physician-diagnosed asthma and current wheezing were determined directly from questions “have you ever been diagnosed with asthma?” and “during the last 12 months, have you experienced attacks of wheezing or whistling breathing?”, respectively. The secondary dependent variables of current chest tightness and shortness of breath were determined directly from questions “during the last 12 months, have you experienced a feeling of tightness in your chest?” and “during the last 12 months, have you had an attack of shortness of breath that came on without obvious cause when you were not exercising or doing some physical activity?”, respectively.

### 2.4. Independent Variables: Psychological and Other Factors

All of the information on the independent variables was collected via self-report directly from the participants. Eleven independent variables were selected a priori for evaluations. They were of demographic (age, sex, obesity, urban/rural), socioeconomic (household-spending, education), environmental (current smoking, floor type, cooking fuel), and psychological (diagnosed depression, 30-day anxiety) natures (for more details see Table 3). The diagnosed depression was derived from the question “have you ever been diagnosed with depression?” and 30-day anxiety from the question “overall in the last 30 days, how much of a problem you have with worry or anxiety?” Since the short questionnaire of WHS did not capture all 11 variables, only those countries (45 for diagnosed asthma and 44 for current wheezing) which used the long questionnaire were analyzed using multiple logistic regressions. Purposeful selection method was used to generate the final models, in which, age and sex were always retained for their biological importance and confounding variables, defined as altering the odds ratios (ORs) of any statistically significant variables by more than 15%, were considered [15]. Self-reported diagnosed depression and 30-day anxiety were included in all the multiple logistic regressions in order to assess the strength and consistency of the associations between diagnosed asthma and current wheezing with diagnosed depression and 30-day anxiety.

### 2.5. Statistical Analysis

All the statistical analyses were conducted using STATA 9.0. Normalized individual design weights were accounted for in all analyses to adjust for the multistage sampling design. The samples were nationally representative in all countries except China, Comoros, Cote d'Ivoire, India, and the Russia. Multiple logistic regressions with Taylor linearization were used to assess the association between diagnosed depression/30-day anxiety and diagnosed asthma/current wheezing adjusted for potential confounders [16]. Information on 30-day anxiety did not appear on the short WHS questionnaire; thus only those countries used the long questionnaire were analyzed for the multiple logistic regression.

## 3. Results

### 3.1. Diagnosed Depression and Diagnosed Asthma/Current Wheezing

**Figure 1:**
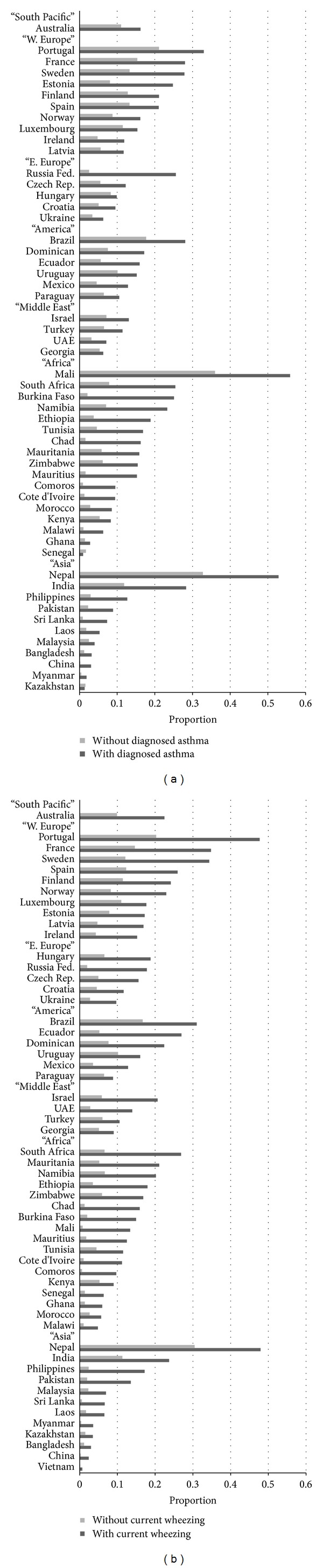
(a) Weighted proportions of diagnosed depression by diagnosed asthma. (b) Weighted proportions of diagnosed depression by current wheezing.

The proportion of respondents who reported of having ever been diagnosed with depression was generally higher (in 52 out of 54 countries) in those who reported of having ever been diagnosed with asthma than those who reported of never being diagnosed with asthma (Figure 1(a)). In the multiple logistic regression, diagnosed depression was significantly associated with diagnosed asthma (*P* < 0.05) in 35 out of 54 countries after controlling for age and sex (Table 1).

The proportion of respondents who reported of having ever been diagnosed with depression was consistently higher (in all 54 countries) in those with current wheezing than those without current wheezing (Figure 1(b)). In the multiple logistic regression, diagnosed depression was significantly associated with current wheezing (*P* < 0.05) in 45 out of 54 countries after controlling for age and sex (Table 2). 

### 3.2. 30-Day Anxiety and Diagnosed Asthma/Current Wheezing

**Figure 2:**
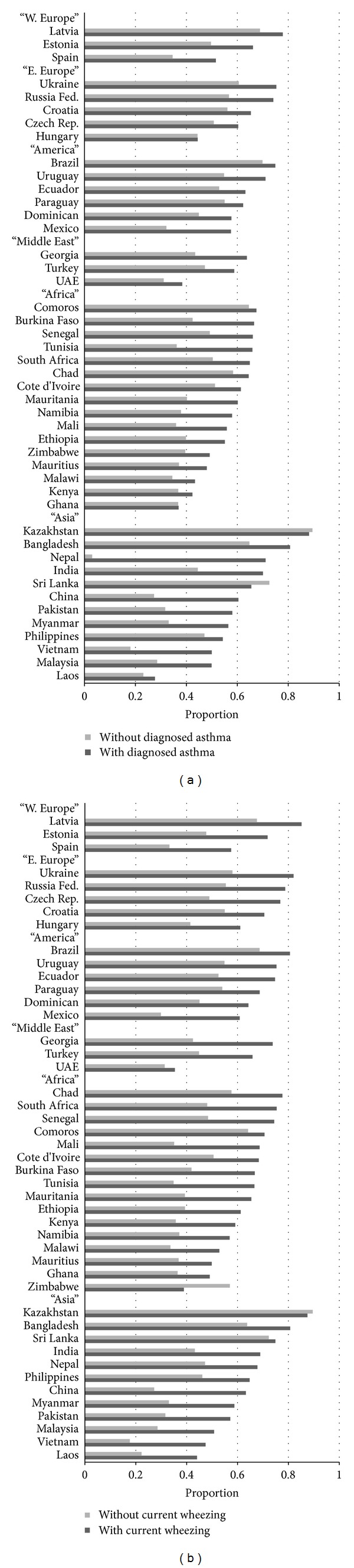
(a) Weighted proportions of 30-day anxiety by diagnosed asthma. (b) Weighted proportions of 30-day anxiety by current wheezing.

The proportion of respondents who reported experiencing some degree of anxiety in the past 30 days was generally higher (in 43 out of 45 countries) in those who had ever been diagnosed with asthma than those without such diagnosis (Figure 2(a)). In the multiple logistic regression, 30-day anxiety was significantly associated with diagnosed asthma (*P* < 0.05) in 18 out of 45 countries and a protective factor in one country (Sri Lanka) after controlling for age and sex (Table 1).

The proportion of respondents who had reported experiencing some degree of anxiety in the past 30 days was generally higher (in 43 out of 45 countries) in those with current wheezing than those without current wheezing (Figure 2(b)). In the multiple logistic regression, 30-day anxiety was significantly associated with current wheezing (*P* < 0.05) in 37 out of 45 countries after controlling for age and sex (Table 2).

### 3.3. Diagnosed Depression/30-Day Anxiety and Current Chest Tightness/Current Shortness of Breath

We also assessed the association between 30-day anxiety and diagnosed depression and two respiratory problems: current chest tightness and shortness of breath (Table 2). In the multiple logistic regression, 30-day anxiety was associated (*P* < 0.05) with current chest tightness and shortness of breath in 41 and 40 out of 45 countries, respectively, after controlling for age and sex. Self-reporting of having been diagnosed with depression was significantly associated with current chest tightness (*P* < 0.05) and shortness of breath (*P* < 0.05) in 48 and 47 out of 54 countries, respectively, after controlling for age and sex.

## 4. Discussions

Our study showed that diagnosed depression and 30-day anxiety were associated with diagnosed asthma and current wheezing in greater than 40% of the countries. The association was particularly strong in some Asian and African countries (Table 1), although some of these countries tended to have low prevalence in both self-reported asthma and depression diagnoses resulting in wide confidence limits. Other respiratory factors suggestive of possible asthma including current chest tightness and current shortness of breath also showed a general pattern of strong and consistent association with diagnosed depression and 30-day anxiety.

The WHS applied multiple psychometric techniques and cultural applicability tests to achieve reliability and cross-cultural validity, such as backtranslation and anchoring vignettes [13]. Factor analysis exploring the cross-cultural comparability of the reporting of respiratory symptoms and clinical definitions of respiratory diseases including asthma showed that symptom patterns were highly comparable in all studied countries who participated in the European Community Respiratory Health Survey (ECRHS) [17]. The authors therefore concluded that cross-cultural variations were insignificant, and unlikely to adversely affect the validity of international comparisons [17]. This supports the cross-cultural validity of the WHS survey because both the WHS and ECRHS used very similar question items and wordings in assessing asthma status and respiratory symptoms in adults. Self-reports of anxiety and depression were generally well accepted for epidemiological surveys [18]. The self-reported Behavioral Risk Factor Surveillance System (BRFSS), by the Centers for Disease Control and Prevention (CDC), used the prior 30 days as a valid period for anxiety symptoms in adults [19]. This period of measurement has been previously shown to be a valid indicator of the current burden of mental distress in patients with common mental disorders. Given the consistency and magnitude of the results, the large number of participated countries, the representative sampling methods employed, and the generally high response rate achieved, the results from this study were considered reliable and robust. 

The USA did not participate in the WHS, partly because it had a well-established National Health Interview Survey (NHIS) on adults [20]. Combining 7 years (2001–2007) of NHIS data, Oraka et al. (2010) examined the relationship between current asthma and serious psychological distress (SPD) defined as scoring 13 or greater in the Kessler Nonspecific Psychological Distress Scale (K-6). Using data from 186,738 adults, the authors found that the prevalence of SPD in current asthmatics was more than doubled (7.5%) that of nonasthmatics (2.6%) [21]. Chun et al. [23] explored the relationship between mental health and asthma in 355,710 American adults using the Behavioral Risk Factor Surveillance System (BRFSS) [22]. Mental health problems were defined based on self-report of the total number of days in the last 30 days that the respondent rated his/her mental health as “not good” due to stress, depression, or problems with emotions. An association between poor mental health and current asthma was found. A dose-response relationship was identified by relative risk ratios (RRR) for current asthma as 1.38, 1.49, 1.67, and 2.75 for “≤1 week”, “1-2 weeks”, “2-3 weeks”, and “≥3 weeks” of poor mental health, respectively, when compared to those who reported not experiencing any degree of poor mental health recently [23]. These two large US studies helped filling the gap from the WHS for North American regions by showing evidence congruent to this current study.

Due to the cross-sectional nature of the WHS, the temporality between asthma, wheezing, depression, and anxiety could not be determined. Nonetheless, results from recent longitudinal studies have suggested that depression and anxiety could act as predisposing factors before someone was diagnosed with asthma [1, 8]. A meta-analysis examining 34 prospective cohort studies indicated that a greater degree of psychosocial stressors was associated with greater incidence of atopic diseases including allergic asthma [24].

The underlying biologic mechanisms between psychological factors and asthma were largely unknown. Some suggested that neuroimmunomodulatory mechanisms were at play. In particular, it was shown that stress acted principally through altered regulation of the hypothalamic-pituitary-adrenal (HPA) axis and sympathetic-adreno-medullary (SAM) nervous system [25]. The subsequent change in levels of neurohormones (and in particular endogenous glucocorticoids) has considerable immunomodulatory effects, including an atopy (or Th2) biased response favoring allergic outcomes including allergic asthma [25].

## 5. Conclusions

The global associations between asthma and psychological conditions have both public health and individual clinical importance. By intervening potential psychological issues, it might be able to prevent the occurrence of some asthma cases. Whereas among asthma patients, properly recognizing the existence of psychological issues could both improve patients' quality of life and prevent further complications from the comorbidity. The comorbidity of depression and asthma could place a compounding financial burden in many nations' health care systems; thus policy makers and other stakeholders should aim at developing effective preventive and clinical strategies to better prevent and manage the conditions among patients and populations as a whole.

## Figures and Tables

**Table 1:** Odds ratios and 95% confident intervals of diagnosed depression and 30-day anxiety on diagnosed asthma and current wheezing in adults by region and country.

	Diagnosed asthma		Current wheezing	
Country*	30-day anxiety	Diagnosed depression	30-day anxiety	Diagnosed depression
	Adjusted OR (95% CI)^†^	Adjusted OR (95% CI)	Adjusted OR (95% CI)	Adjusted OR (95% CI)
South Pacific				
Australia^‡^		**1.5 (1.0–2.3)**		**2.5 (1.7–3.7)**
W. Europe				
Estonia	1.6 (0.8–3.0)	**2.5 (1.4–4.6)**	**2.2 (1.3–3.7)**	**1.8 (1.1–3.1)**
Finland^‡^		**1.8 (1.1–2.9)**		**2.4 (1.5–3.6)**
France^‡^		**2.0 (1.1–3.7)**		**3.1 (1.5–6.3)**
Ireland^‡^		**2.7 (1.3–5.6)**		**4.1 (2.0–8.5)**
Latvia	1.4 (0.5–3.6)	2.0 (0.3–11.5)	**2.4 (1.1–5.2)**	**4.0 (1.8–9.1)**
Luxembourg^‡^		1.4 (0.7–3.1)		1.8 (0.9–3.3)
Norway^‡^		**2.0 (1.1–3.4)**		**3.0 (1.7–5.3)**
Portugal^‡^		**2.0 (1.5–3.3)**		**3.5 (2.1–5.9)**
Spain	**1.9 (1.4–2.5)**	**1.4 (1.0–2.0)**	**2.4 (1.9–3.1)**	**1.8 (1.3–2.4)**
Sweden^‡^		2.6 (0.9–7.8)		**4.2 (2.0–8.7)**
E. Europe				
Croatia	1.3 (0.7–2.4)	1.2 (0.4–3.9)	**1.9 (1.1–3.4)**	1.7 (0.7–4.2)
Czech Rep.	1.2 (0.5–2.9)	2.0 (0.7–5.8)	**3.1 (1.5–6.8)**	1.7 (0.7–4.3)
Hungary	1.1 (0.6–1.8)	1.4 (0.7–3.2)	**1.8 (1.3–2.6)**	**2.5 (1.6–3.8)**
Russia	1.2 (0.7–2.2)	**10.4 (2.0–53.4)**	**1.9 (1.0–3.6)**	**8.4 (3.1–22.9)**
Ukraine	**1.9 (1.0–3.4)**	1.6 (0.8–3.3)	**2.9 (2.0–4.3)**	**3.3 (1.8–6.0)**
America				
Brazil	1.2 (1.0–1.5)	**1.8 (1.4–2.3)**	**1.7 (1.4–2.2)**	**2.1 (1.7–2.5)**
Dominican	**1.4 (1.0–1.9)**	**2.0 (1.3–3.1)**	**1.8 (1.1–2.8)**	**2.5 (1.4–4.2)**
Ecuador	1.3 (0.7–2.7)	**3.0 (1.6–5.7)**	1.9 (0.9–3.8)	**4.9 (2.6–9.3)**
Mexico	**2.5 (2.1–3.0)**	**1.9 (1.4–2.5)**	**2.9 (2.4–3.6)** ^§^	**2.5 (1.8–3.3)** ^§^
Paraguay	1.1 (0.8–1.6)	1.4 (0.8–2.4)	**1.8 (1.4–2.3)**	1.2 (0.7–1.8)
Uruguay	**2.0 (1.6–2.6)**	1.3 (0.8–2.3)	**2.4 (1.7–3.5)**	**1.5 (1.0–2.2)**
Middle East				
Georgia	1.6 (0.8–3.5)	0.9 (0.4–2.1)	**2.9 (1.8–4.7)**	1.3 (0.7–2.4)
Israel^‡^		**2.0 (1.1–4.0)**		**3.9 (2.2–6.9)**
Turkey	1.3 (0.99–1.8)	1.5 (1.0–2.1)	**2.2 (1.9–2.6)**	**1.4 (1.1–1.8)**
UAE	1.3 (0.8–2.3)	**2.7 (1.1–6.8)**	0.9 (0.3–2.2)	**6.2 (1.2–31.8)**
Africa				
Burkina Faso	**2.0 (1.2–3.6)**	**13.0 (5.6–30.1)**	**2.2 (1.4–3.5)**	**7.3 (3.3–15.8)**
Chad	1.0 (0.7–1.6)	**9.1 (3.9–21.3)**	**2.4 (1.6–3.6)**	**12.8 (6.6–24.6)**
Comoros	1.1 (0.7–1.8)	**18.4 (5.2–65.6)**	1.2 (0.8–1.8)	**16.0 (5.4–47.9)**
Cote d'Ivoire	1.4 (0.9–2.1)	**6.1 (2.5–15.1)**	**1.9 (1.2–2.9)**	**10.0 (5.2–19.4)**
Ethiopia	1.5 (0.9–2.5)	**5.0 (2.7–9.3)**	**1.8 (1.3–2.5)**	**4.8 (3.1–7.4)**
Ghana	0.9 (0.6–1.3)	2.0 (0.7–5.2)	1.4 (0.9–2.3)	**3.9 (1.4–11.1)**
Kenya	1.1 (0.6–2.3)	1.5 (0.6–3.6)	**2.4 (1.5–3.9)**	1.2 (0.6–2.7)
Malawi	**1.4 (1.0–1.9)**	**5.5 (2.7–11.1)**	**2.3 (1.7–3.1)**	**4.4 (2.1–9.5)**
Mauritania	1.2 (0.8–1.7)	**7.3 (3.4–16.0)**	1.4 (0.9–1.9)	**5.3 (2.6–11.0)**
Mauritius	**1.8 (1.2–2.8)**	**2.1 (1.4–3.4)**	**2.3 (1.7–3.2)**	**3.3 (2.1–5.0)**
Morocco	n/a^§^	**2.9 (1.4–6.2)**	n/a^§^	**2.0 (1.0–3.8)**
Namibia	1.6 (0.99–2.7)	**3.5 (2.0–6.1)**	**1.9 (1.4–2.6)**	**2.9 (1.9–4.4)**
Senegal	**1.9 (1.1–3.3)**	0.5 (0.1–1.9)	**2.7 (1.6–4.5)**	**5.9 (2.0–17.6)**
S. Africa	1.5 (0.99–2.2)	**3.3 (1.6–6.9)**	**2.4 (1.7–3.4)**	**4.1 (2.5–6.7)**
Tunisia	**2.5 (1.6–4.0)**	**4.5 (2.6–7.7)**	**3.1 (2.2–4.3)**	**2.2 (1.3–3.7)**
Zimbabwe	1.1 (0.7–1.7)	**2.5 (1.0–6.0)**	**1.6 (1.1–2.2)**	**2.7 (1.5–4.7)**
Asia				
Bangladesh	**2.0 (1.3–3.2)**	**2.1 (1.1–4.1)**	**2.1 (1.6–3.0)**	**2.2 (1.3–3.9)**
China	**3.3 (1.8–6.0)**	5.1 (0.7–36.7)	**3.4 (2.2–5.3)**	4.2 (0.5–33.0)
India	**1.9 (1.4–2.6)**	**2.1 (1.5–2.7)**	**2.2 (1.6–2.9)**	**1.7 (1.4–2.2)**
Kazakhstan	0.9 (0.4–2.1)	0.8 (0.2–2.8)	0.9 (0.5–1.6)	1.9 (0.7–5.1)
Laos	1.0 (0.7–1.6)	**3.7 (1.6–8.1)**	**2.3 (1.6–3.2)**	**3.6 (1.8–6.9)**
Malaysia	**2.5 (1.9–3.2)**	1.2 (0.6–2.1)	**2.6 (2.0–3.3)**	**2.5 (1.5–4.0)**
Myanmar	**2.4 (1.7–3.6)**	2.4 (0.5–11.5)	**2.5 (1.6–4.0)**	**5.4 (1.9–15.4)**
Nepal	**1.5 (1.1–2.1)**	**1.9 (1.4–2.5)**	**1.7 (1.5–2.0)**	**1.8 (1.5–2.0)**
Pakistan	**2.4 (1.8–3.3)**	**2.7 (1.4–5.5)**	**2.2 (1.6–3.0)**	**6.0 (3.2–11.1)**
Philippines	1.1 (0.9–1.3)	**4.6 (3.2–6.5)**	**1.6 (1.3–1.9)**	**7.2 (5.3–9.8)**
Sri Lanka	**0.6 (0.5–0.9)**	**7.1 (3.2–16.0)**	1.1 (0.8–1.4)	**12.2 (6.5–22.7)**
Vietnam	**4.0 (2.0–8.2)**	n/a^§^	**3.2 (1.9–5.3)**	5.0 (0.5–46.9)^§^

*Unless specified otherwise, ORs are derived from the final multiple logistic models containing age, sex, 30-day anxiety, diagnosed depression, significant (*P* < 0.05) variables, and confounders. ^†^Bolded ORs are statistically significant at *P* < 0.05. ^‡^30-day anxiety is unavailable due to the use of short WHS questionnaire; ORs are adjusted for age and sex. ^§^Missing >25% of data.

**Table 2:** Odds ratios and 95% confident intervals of diagnosed depression and 30-day anxiety on current chest tightness and current shortness of breath in adults by region and country.

	Current chest tightness		Current shortness of breath	
Country*	30-day anxiety	Diagnosed depression	30-day anxiety	Diagnosed depression
	Adjusted OR (95% CI)^†^	Adjusted OR (95% CI)	Adjusted OR (95% CI)	Adjusted OR (95% CI)
South Pacific				
Australia^‡^		**3.5 (2.6–4.8)**		**3.3 (2.2–4.9)**
W. Europe				
Estonia	**4.4 (2.9–6.6)**	**1.7 (1.2–2.6)**	**2.3 (1.4–3.6)**	**2.3 (1.1–4.6)**
Finland^‡^		1.5 (0.8–2.6)		3.1 (1.7–5.5)
France^‡^		**3.8 (2.0–7.1)**		**3.8 (1.6–9.1)**
Ireland^‡^		**8.7 (4.4–16.9)**		**9.4 (4.4–20.0)**
Latvia	**5.1 (2.6–10.2)**	**4.7 (2.5–9.0)**	**2.3 (1.1–5.0)**	**6.2 (3.0–13.1)**
Luxembourg^‡^		**3.9 (2.2–7.1)**		**3.7 (1.8–7.4)**
Norway^‡^		**2.5 (1.5–4.2)**		1.6 (0.6–4.3)
Portugal^‡^		**3.5 (2.3–5.4)**		**4.0 (2.7–5.7)**
Spain	**3.7 (2.8–4.9)**	**1.9 (1.5–2.5)**	**3.3 (2.4–4.6)**	**2.1 (1.6–2.9)**
Sweden^‡^		**2.5 (1.3–4.8)**		**3.0 (1.1–8.6)**
E. Europe				
Croatia	**2.2 (1.4–3.4)**	**2.3 (1.2–4.5)**	**2.2 (1.3–3.8)**	**3.6 (1.7–7.8)**
Czech Rep.	**5.0 (2.3–10.9)**	0.8 (0.3–1.8)	1.6 (0.7–3.8)	**4.7 (1.7–13.4)**
Hungary	**2.2 (1.5–3.0)**	**2.2 (1.4–3.3)**	**2.5 (1.8–3.5)**	**3.3 (2.2–5.2)**
Russia	**1.8 (1.1–2.8)**	**4.6 (1.7–12.3)**	**2.1 (1.3–3.6)**	**6.8 (2.1–21.5)**
Ukraine	**2.9 (2.1–4.1)**	**2.9 (1.6–5.1)**	**3.5 (2.0–6.2)**	**3.1 (1.9–5.4)**
America				
Brazil	**2.6 (2.1–3.2)**	**2.3 (1.9–2.7)**	**2.6 (2.0–3.5)**	**2.3 (1.9–2.9)**
Dominican	**2.2 (1.6–3.1)**	**2.4 (1.7–3.4)**	**2.6 (1.5–4.6)**	**3.4 (1.9–6.0)**
Ecuador	**2.3 (1.4–3.6)** ^§^	**4.0 (2.7–5.9)** ^§^	1.9 (0.9–3.9)^§^	**4.0 (2.2–7.2)** ^§^
Mexico	**3.3 (2.9–3.8)** ^§^	**3.3 (2.5–4.3)** ^§^	**3.7 (3.1–4.5)** ^§^	**3.3 (2.6–4.3)** ^§^
Paraguay	**2.6 (2.1–3.3)**	**1.9 (1.3–2.7)**	**2.7 (2.0–3.5)**	**2.9 (2.0–4.1)**
Uruguay	**2.7 (1.7–4.4)**	**3.2 (2.7–3.9)**	**2.9 (2.1–4.1)**	**3.6 (2.4–5.2)**
Middle East				
Georgia	**2.4 (1.6–3.5)**	**1.8 (1.3–2.6)**	**2.6 (1.8–3.8)**	2.0 (0.9–4.5)
Israel^‡^		**4.2 (2.5–7.0)**		**3.1 (1.7–5.7)**
Turkey	**2.4 (2.0–2.8)**	**1.5 (1.2–1.8)**	**2.2 (1.7–2.9)**	**2.2 (1.6–3.0)**
UAE	**3.1 (1.6–6.2)**	**6.7 (2.8–16.4)**	**2.7 (1.0–7.3)**	**5.4 (2.2–13.4)**
Africa				
Burkina Faso	**3.1 (2.1–4.6)**	**9.5 (4.5–20.1)**	**3.6 (2.5–5.1)**	**10.2 (4.4–23.4)**
Chad	**2.5 (1.8–3.5)**	**13.1 (6.5–26.2)**	**3.2 (2.0–5.1)**	**24.1 (11.4–50.9)**
Comoros	1.5 (0.99–2.4)	**7.1 (2.4–20.9)**	**1.7 (1.0–3.0)**	**10.5 (3.4–32.5)**
Cote d'Ivoire	**3.5 (2.3–5.2)**	**6.6 (3.1–14.0)**	**2.8 (1.8–4.3)**	**9.7 (4.5–20.8)**
Ethiopia	**2.6 (1.9–3.5)**	**4.8 (3.0–7.6)**	**2.4 (1.7–3.4)**	**5.9 (3.7–9.4)**
Ghana	**2.0 (1.3–3.1)**	**3.0 (1.0–8.5)**	**1.7 (1.1–2.6)**	**3.5 (1.2–10.6)**
Kenya	**2.8 (1.8–4.5)**	1.0 (0.5–2.3)	**2.2 (1.5–3.1)**	1.2 (0.5–2.9)
Malawi	**2.7 (2.1–3.6)**	**4.2 (2.2–8.3)**	**2.6 (1.9–3.5)**	**6.8 (3.2–14.6)**
Mauritania	**1.6 (1.2–2.1)**	**12.4 (7.1–21.6)**	**1.6 (1.1–2.2)**	**23.0 (12.3–43.0)**
Mauritius	**1.9 (1.3–2.9)**	**4.2 (2.6–6.8)**	**2.7 (1.6–4.5)**	**4.3 (2.6–7.0)**
Morocco	n/a^§^	**3.0 (1.8–5.0)**	n/a^§^	**3.1 (1.8–5.4)**
Namibia	**3.8 (2.7–5.2)**	**3.1 (2.0–4.7)**	**2.3 (1.6–3.4)**	**4.5 (2.9–7.1)**
Senegal	**3.8 (2.6–5.6)** ^§^	2.2 (0.8–6.1)^§^	**2.5 (1.7–3.8)** ^§^	**3.7 (1.5–9.2)** ^§^
S. Africa	**2.5 (1.8–3.3)**	**3.0 (1.7–5.3)**	**2.4 (1.6–3.5)**	**3.6 (1.9–7.0)**
Tunisia	**4.0 (3.1–5.3)**	**1.6 (1.0–2.4)**	**3.2 (2.2–4.6)**	**3.1 (2.0–4.9)**
Zimbabwe	**2.0 (1.5–2.6)**	**2.3 (1.5–3.6)**	**1.8 (1.3–2.4)**	**2.4 (1.5–4.1)**
Asia				
Bangladesh	**3.1 (2.3–4.2)**	**4.0 (2.5–6.6)**	**2.8 (1.9–4.2)**	**2.9 (1.6–5.1)**
China	**5.3 (2.8–9.7)**	**5.5 (1.3–23.8)**	**4.8 (2.4–9.3)**	4.5 (0.6–33.8)
India	**4.0 (3.1–5.1)**	**2.0 (1.6–2.6)**	**3.1 (2.2–4.4)**	**2.6 (2.0–3.4)**
Kazakhstan	1.0 (0.7–1.4)	2.3 (0.9–6.4)	0.8 (0.6–1.2)	2.4 (0.8–7.0)
Laos	**2.1 (1.6–2.9)**	**3.4 (1.8–6.5)**	**2.5 (1.5–3.9)**	**4.8 (2.5–9.2)**
Malaysia	**3.8 (3.0–4.8)**	**2.8 (1.6–4.7)**	**4.1 (2.8–6.0)**	**2.4 (1.6–4.7)**
Myanmar	**3.5 (2.3–5.3)**	**6.0 (2.6–13.6)**	**4.4 (3.0–6.6)**	**7.5 (2.9–20.0)**
Nepal	**2.1 (1.8–2.5)**	**2.1 (1.8–2.4)**	**1.5 (1.3–1.9)**	**1.8 (1.5–2.1)**
Pakistan	**3.7 (2.8–5.0)**	**3.9 (2.2–6.8)**	**2.8 (1.7–4.5)**	**4.4 (2.4–7.9)**
Philippines	**1.8 (1.5–2.1)**	**7.5 (5.5–10.2)**	**1.9 (1.5–2.5)**	**8.6 (6.1–12.3)**
Sri Lanka	0.9 (0.6–1.3)	**6.4 (3.2–12.9)**	1.0 (0.8–1.4)	**8.1 (3.4–19.1)**
Vietnam	**6.5 (3.9–10.7)**	2.0 (0.3–16.9)	**6.0 (2.9–12.5)**	5.1 (0.9–28.3)

*All ORs are derived from the final multiple logistic models containing age, sex, 30-day anxiety, and diagnosed depression. ^†^Bolded ORs are statistically significant at *P* < 0.05. ^‡^30-day anxiety is unavailable due to the use of short WHS questionnaire; ORs are adjusted for age and sex. ^§^Missing >25% of data.

**Table 3:** Summary of independent variables.

Variable		Recoded responses*
	Demographic	

Sex		(i) Male
		(ii) Female
Age group		(i) 18–34 years
		(ii) 35–54 years
		(iii) ≥55 years
Obesity		(i) BMI < 30
		(ii) BMI ≥ 30
Urban/rural		(i) Rural setting, residence
		(ii) Urban setting, residence

	SES indicator	

Household spending		(i) Lower 50%ile, survey sample
		(ii) Upper 50%ile, survey sample
Education		(i) Before the completion of secondary school
		(ii) Secondary school completed or beyond

	Psychological	

30-day anxiety		(i) Did not experience anxiety at all in last 30 days
		(ii) Experienced at least some anxiety in last 30 days
Diagnosed depression		(i) No (never been diagnosed)
		(ii) Yes (has been diagnosed)

	Environmental	

Current smoking		(i) No
		(ii) Yes
Floor type		(i) Earth floor, dwelling
		(ii) Hard floor, dwelling
Cooking fuel		(i) Other
		(ii) Hydrocarbon (gas, kerosene, coal, and charcoal)

*Reference groups are indicated with (i).
